# Mentors' resources and premature match closure in challenging contexts: testing a model of mediating processes in an online and a school-based mentoring program

**DOI:** 10.3389/fpsyg.2025.1559060

**Published:** 2025-08-29

**Authors:** Sonja Bayer, Heidrun Stoeger, Albert Ziegler

**Affiliations:** ^1^Department of School Research, School Development, and Evaluation, University of Regensburg, Regensburg, Germany; ^2^Department of Psychology, University of Erlangen Nuremberg, Erlangen, Germany

**Keywords:** mentoring, premature match closure, online mentoring, helplessness, confidence in own ability, beliefs, COVID-19

## Abstract

**Introduction:**

This research aimed to investigate the impact of mentors' resources on the premature termination of mentoring relationships in challenging contexts as well as mediating psychological processes.

**Method:**

In two studies, we analyzed the data of 98 mentors from an online mentoring program for girls in STEM subjects and the data of 60 mentors from a school-based mentoring program with talented youth. Participants were surveyed with a standardized questionnaire about their mentoring experiences during the first peak of the COVID-19 pandemic in Germany, which created a challenging context.

**Results:**

The results indicated that a reduction of mentoring resources is associated with an increased risk of premature match closure. This effect of resources was mediated in two ways: by (a) mentors' confidence in their mentoring abilities, which predicted feelings of helplessness, and (b) mentors' beliefs in the modifiability of deficits and the stability of abilities, which predicted adaptive responses to failure. Furthermore, mentors in the school-based program reported a more significant reduction in mentoring resources, which, in turn, was more strongly associated with premature match closure than in the online mentoring program.

**Discussion:**

This suggests that online mentoring might be more robust than face-to-face mentoring under unfavorable environmental conditions. Overall, our study points to equipping mentors with adequate resources and offering ongoing support, especially in challenging environments.

## 1 Introduction

Mentoring is often defined as a relatively stable dyadic relationship between one or more experienced individuals (mentors) and one or more less experienced individuals (mentees), characterized by mutual trust, goodwill, and the shared goal of the mentee's advancement and development (Stoeger et al., 2009). It can be a highly effective way to support individuals' personal, academic, or professional development (Bloom, 1985; Eby et al., 2008; Subotnik et al., 2021). Mentoring can occur in various contexts and can be carried out in different formats (Mullen and Klimaitis, 2021). It is also widely used to support young people in various development-related areas (DuBois et al., 2002; Raposa et al., 2019b). Youth can benefit from mentoring relationships in a wide range of outcomes, including educational, social, psychological, and health domains (Raposa et al., 2019b).

The benefits of mentoring for youth increase as the duration of the relationship increases (Grossman et al., 2012; Grossman and Rhodes, 2002; Karcher, 2005; Herrera et al., 2011). For example, in a study by (Grossman and Rhodes 2002), positive mentoring outcomes in youth mentoring were most significant when the mentee-mentor relationship lasted at least 12 months. In contrast, mentees in relationships that ended prematurely (i.e., 3 to 6 months of a year-long program) showed poorer outcomes, and mentees in relationships that ended within the first 3 months of mentoring even experienced adverse outcomes (e.g., a decline in self-esteem and academic competence) compared to a control group that did not receive mentoring.

The premature termination of the mentoring relationship is referred to in the literature as premature match closure (Kupersmidt et al., 2017c). Due to the possible negative effects (Grossman and Rhodes, 2002; Grossman et al., 2012) and its frequent occurrence, premature match closure is a significant problem in mentoring practice. For example, studies show that between one-third and more than half of mentoring relationships are terminated prematurely (Grossman and Rhodes, 2002; DeWit et al., 2016; Keller and Spencer, 2018). A more comprehensive understanding of the causes and underlying mechanisms of premature match closure is important to maximize mentoring's positive effects and avoid its negative effects.

Previous research on premature match closure has identified reasons and mechanisms at the participant (mentor and mentee), context, and program levels. On the mentee level, associations were found between early match closure and age, gender, at-risk status, level of interest or satisfaction with mentoring, networking (in online mentoring), and program adherence (Grossman et al., 2012; Grossman and Rhodes, 2002; Kupersmidt et al., 2017c; Spencer et al., 2020; Uebler et al., 2023; Stelter et al., 2018; Schwartz et al., 2011). On the mentor level, correlations between premature match closure and income, age, marital status, gender, previous mentoring experience, and the degree of mentor preparation were demonstrated (Grossman and Rhodes, 2002; Herrera et al., 2011; Kupersmidt et al., 2017c; McQuillin and Lyons, 2021; Uebler et al., 2023). At the context level, correlations were found between early match closure and life circumstances (e.g., lack of time or moving) or parental influence on youth mentoring (DeWit et al., 2016; Keller and Spencer, 2018; Spencer et al., 2020). At the program level, associations were found between early match closure and the number of implemented research-based critical factors for successful mentoring in programs (Kupersmidt et al., 2017b; Stelter et al., 2018), frequency and steadiness of mentee-mentor contact, mentor-mentee matching, and participant training and support (DeWit et al., 2016; McQuillin and Lyons, 2021; Spencer et al., 2020; Uebler et al., 2023). Thus, numerous studies at different levels (individual, contextual, program level) draw a highly complex picture of premature match closure. However, a neglected field of research are the psychological mechanisms underlying premature match closure on the level of mentors and the necessary resources for a longer-term mentoring relationship. Therefore, a precise understanding of these psychological mechanisms at the mentor level is essential to prevent premature match closure and to support mentors adequately in their mentoring. Especially in challenging times, a better understanding of these mechanisms could help to support mentors in a targeted way and provide adequate resources. Our study aimed to test a theoretical model that systematically considers both resources and psychological mechanisms at the mentor level and to test it empirically in two studies.

## 2 Theoretical background

### 2.1 Resources in mentoring

Mentoring research suggests that resources play an essential role in the successful implementation of mentoring (Stoeger et al., 2021). From a systemic view, the success of mentoring episodes depends on the resources within mentees and mentors as well as resources in the environment (Ziegler et al., 2021b). Due to their regulative power in shaping the mentoring process, mentors' resources are especially likely to play an outstanding role.

A framework that suits well to categorize and comprehensively capture mentors' resources is the educational and learning capital approach, which incorporates environmental and internal resources (Ziegler, 2005). To cover the entirety of an individual and their material, social, and informational environments, the model postulates 10 different types of resources (referred to as capitals), of which five are external resources in the direct environment of an individual and five are internal resources located inside the individual itself. Numerous studies have shown that the amount of external and internal resources that are available to learners predicts their academic development (Leana-Taşcilar, 2015; Ziegler et al., 2019; Vladut et al., 2013, 2015).

Mentoring research offers ample evidence that mentors' different types of external and internal resources influence the development of mentoring relationships. In a study by Grossman and Rhodes (2002), mentors with lower incomes and mentors who had experienced emotional, sexual, or physical abuse were most likely to be in early terminating relationships. On the other hand, enhanced mentor training and support through the mentoring agency increase mentors' plans to continue mentoring (McQuillin et al., 2015). Further, if parents of mentees are highly supportive of their child's adult mentor, mentoring relationships are less likely to end prematurely (DeWit et al., 2016). An example of mentors' internal resources is their previous mentoring experiences: if mentors have much experience in mentoring; the risk of premature match closure is reduced (Uebler et al., 2023).

Challenging situations in which resources are restricted can have negative consequences in terms of psychological processes and behavior. It has been shown that a lack of job resources among employees is associated with burnout and lower work engagement (Crawford et al., 2010). University students' perceived social resources at university are linked to their institutional commitment, suggesting that low social resources increase the risk of dropping out of university (McEwan, 2013). Families facing economic hardship, such as low income or negative economic events, experience economic pressure that negatively impacts family functioning and, ultimately, a range of developmental outcomes for adults and children (Conger et al., 2010). Accordingly, environmental changes associated with limiting mentoring resources can be assumed to result in dysfunctional mentor behavior and thereby increase the risk of premature match closure.

### 2.2 Two psychological processes explaining premature match closure in challenging times

We expect two psychological processes at the mentor level that are triggered in challenging situations with limited resources: a decrease in mentors' confidence in their mentoring abilities on the one hand, and a change in their beliefs about the nature of their abilities on the other hand.

Mentors' confidence in their mentoring abilities is a key psychological concept in mentoring research: how competent mentors think they act as a mentor determines the development and results of mentoring relationships (Raposa et al., 2016; Karcher et al., 2005; Ferro et al., 2013; Parra et al., 2002). On the other hand, mentors' perceptions of their abilities are influenced by external factors: it has been shown that the amount and quality of perceived training increase mentors' confidence in their abilities (Parra et al., 2002; Kupersmidt et al., 2017a).

Low confidence in one's abilities and skills in a specific domain can lead to feelings of helplessness (Ziegler et al., 2005b), which has negative consequences on a behavioral, motivational, and emotional level which—for example—becomes evident in lower academic achievement among students (Filippello et al., 2020). Therefore, decreased mentors' self-evaluations in challenging situations are likely to be transmitted into maladaptive behavior in mentoring (e.g., reduced contact initiatives, fewer initiatives to foster their mentees' development by setting challenges for them) through feelings of helplessness that they experience in the face of a reduction of their resources. This maladaptive behavior—in the worst case—might lead to premature closure of mentoring relationships.

As a second process, we assume that mentors' beliefs regarding their mentoring abilities mediate the relationship between mentors' resources in challenging contexts and mentoring outcomes. Dweck (1999) introduced the concept of implicit theories (also known as mindsets), which describe the extent to which individuals believe that traits and abilities are changeable. Her theory postulates that people with a growth mindset believe their abilities can change with practice, while people with a fixed mindset believe their abilities cannot be changed. It has been shown that implicit theories of ability are related to self-regulatory processes, academic achievement, and mental health outcomes (Costa and Faria, 2018; Burnette et al., 2013, 2020).

Dweck's original theory was refined in the framework of implicit personality theories on modifiability and stability (Ziegler et al., 2010; Ziegler and Stoeger, 2010). In contrast to Dweck's conceptualization of fixed vs. growth mindsets, the extended framework assumes that stability beliefs do not necessarily lead to negative consequences but can be adaptive if these beliefs concern the stability of existing abilities. However, the modifiability component in the extended framework refers to individual deficits. Surveys among students in different countries showed that the extension of Dweck's original theory contributed to improvements in predictions of different indicators of adaptive learning behavior (Ziegler and Stoeger, 2010; Ziegler et al., 2010).

Implicit theories of ability affect cognitive processes and behavior, especially in challenging situations such as ego threat or academic failure (Burnette et al., 2013; Tao et al., 2022; Robins and Pals, 2010). Mentors' beliefs on the nature of their mentoring abilities are likely to play a special role in challenging situations. Specifically, reduced mentoring resources might decrease mentors' beliefs in the modifiability of their deficits and the stability of their abilities in their mentoring practice. These doubts may lead to a maladaptive response to failure and, in turn, increase the risk of premature match closure.

## 3 Present research

The present research aims to explore the role of mentoring resources and psychological processes at the mentor level to explain the premature termination of mentoring relationships, as this is one of the main problems in mentoring practice. We propose a model of antecedents of premature match closure under limited resources based on three main assumptions. First, we expect that the resources available to mentors influence the length of mentoring relationships and, thus, the probability of a premature match closure.

Second and third, we assume two mechanisms that mediate the impact of mentors' resources on premature match closure. Based on previous mentoring research (Parra et al., 2002; Raposa et al., 2016), we assume that mentors' resources influence how mentors perceive their mentoring abilities, leading to feelings of helplessness and behavioral consequences that make premature endings of mentoring relationships more likely. Further, we transfer the framework of implicit personality theories on modifiability and stability (Ziegler and Stoeger, 2010) to the mentoring context and expect mentors' beliefs on the nature of their abilities to be negatively influenced in situations characterized by limited resources. This leads to a maladaptive reaction to failure, which we assume to be precursive of premature match closure.

We conducted two studies among mentors in two different mentoring programs to test our model. We chose to do our research during the COVID-19 pandemic. This created a challenging context where resources were generally restricted due to governmental regulations and social distancing, which negatively affected individuals' lives (Marroquín et al., 2020). Research on mentoring during the COVID-19 pandemic suggests that mentors were also challenged more during that time, and the regulations affected mentoring in a negative sense (Kodele and Rape Žiberna, 2023; Kaufman et al., 2022). In line with this research, we expect that the restrictions in daily life also diminish the resources that mentors need to carry out their mentoring successfully.

## 4 Study 1

### 4.1 Study setting and participants

The first study was conducted among female mentors in the Germany-wide online mentoring program CyberMentor. The program provides girls between the ages of 12 and 18 with at least 1 year of one-on-one interaction with a personal female mentor who is majoring in a STEM subject or working in a STEM field. Mentoring takes place via a protected online platform, including mail, chat, and forum, and is supported by a wide range of information on STEM, study, and career choice. The program is continuously evaluated and optimized based on the results of the accompanying research (Stoeger et al., 2020, 2013, 2016). We invited 411 mentors who started online mentoring in October or December 2019 to participate in the study. After sending three reminders, 107 mentors completed the online questionnaire, which resulted in a 26% response rate. We removed nine participants from this sample as they indicated they had interrupted the mentoring before the COVID-19 pandemic, resulting in a final sample of 98 female mentors (*M*_*age*_ = 31.94, *SD*_*age*_ = 8.74, range = 21 to 61).

### 4.2 Measures

Unless specified otherwise, all items in the questionnaire were rated using a 6-point Likert-type scale, ranging from 1 (*I disagree completely*) to 6 (*I agree completely*). To capture the specific situation and reactions of mentors during the first peak of the COVID-19 pandemic, the items were formulated to relate to the mentoring experience in this period. The questionnaire was introduced with the following note: “The questions refer to the last school year during the pandemic, i.e., from the time of the school closures (during the week beginning March 16, 2020) to the end of the 2019/2020 school year (depending on the German federal state the mentees lived in the school year ended between June 24 and July 29).” Internal consistencies for all scales were good (see Table 1).

**Table 1:** Means and standard deviations, alphas, and intercorrelations on all measures for Study 1 and Study 2.

**Measures**	***M***	***SD***	**α**	**1**	**2**	**3**	**4**	**5**	**6**
*M*				3.48	3.65	4.18	2.89	3.97	–
*SD*				0.80	1.24	0.83	0.99	0.75	–
Cronbach's α				0.80	0.87	0.83	0.88	0.73	–
1. Mentors' resources	4.11	0.94	0.87	–	0.68^**^	0.62^**^	−0.50^**^	0.39^**^	−0.50^**^
2. Confidence in abilities	4.30	1.43	0.94	0.74^**^	–	0.66^**^	−0.59^**^	0.39^**^	−0.44^**^
3. Modifiability and stability beliefs	4.73	0.97	0.93	0.73^**^	0.68^**^	–	−0.56^**^	0.51^**^	−0.42^**^
4. Helplessness	2.64	1.02	0.92	−0.37^**^	−0.34^**^	−0.27^**^	–	−0.46^**^	0.48^**^
5. Adaptive failure response	3.91	0.81	0.84	0.56^**^	0.40^**^	0.63^**^	−0.19	–	−0.47^**^
6. Premature match closure^a^	–	–	–	−0.27^**^	−0.16	−0.22^*^	0.32^**^	−0.18	–

Study 1 (N = 98) is below the diagonal; Study 2 (N = 60) is above the diagonal.

^a^dichotomous variable, shown is the point-biserial correlation with other measures.

^*^p < 0.05; ^**^p < 0.01.

#### 4.2.1 Mentors' resources

To comprehensively assess mentors' resources, we used a shortened and adapted version of the Questionnaire of Educational and Learning Capital (Vladut et al., 2013). The original questionnaire contains five subscales to measure external resources and five subscales to measure internal resources, each consisting of five items. The questionnaire had been adapted to different settings and domains in the past, and also shorter versions of the questionnaire had shown good reliabilities (Reutlinger et al., 2020; Ziegler et al., 2019, 2021a). In our study, the questionnaire was reduced to 10 items and adapted to mentoring during the COVID-19 pandemic. A sample item reads, “Even during the pandemic, I got mentoring support from others when I needed it.”

#### 4.2.2 Confidence in abilities

To assess mentors' confidence in their mentoring abilities, a four-item scale by Dweck and Henderson (1988) was adapted to the context of mentoring during the COVID-19 pandemic. Each item consists of two statements corresponding to a positive self-assessment and a negative self-assessment at the two poles of a six-point Likert scale. A sample item reads, “I trusted myself to provide good mentoring during the pandemic as well.” vs. “I'm not very confident that I can provide good mentoring during the pandemic.”

#### 4.2.3 Modifiability and stability beliefs

A combination of two scales developed by Ziegler and Stoeger (2010) was used to assess mentors' modifiability and stability beliefs. The original scales on the modifiability of ability deficits and the stability of existing abilities were aggregated to one as the factor analysis with the current data did not indicate a two-factor solution. The intercorrelation among both scales was high (*r* = 0.74). Besides adapting the items to the mentoring field during the COVID-19 pandemic, we shortened the scales from 12 to eight items. The items assess mentors' judgments of the stability of their abilities and the modifiability of their ability deficits in mentoring during the pandemic. Sample items are “I can learn a lot in the pandemic when it comes to mentoring.” and “My strengths in pre-pandemic mentoring are still effective during the pandemic.”

#### 4.2.4 Helplessness

To measure the degree of mentors' helplessness during the COVID-19 pandemic, we used an adapted version of a six-item scale developed by Ziegler et al. (2005a). A sample item of the reformulated scale reads, “It didn't matter how committed I was to my mentoring during the pandemic—I still didn't have good success.”

#### 4.2.5 Adaptive failure response

How mentors reacted to failure while mentoring during the COVID-19 pandemic was assessed with an adapted scale developed by Ziegler et al. (2000). The instrument consists of five items and measures the degree to which a person uses the feedback of failure to improve their learning process, for example, by enhancing effort. A sample item reads: “If something in mentoring went wrong during the pandemic, I have tried harder to improve myself.”

#### 4.2.6 Premature match closure

To determine whether mentoring dyads prematurely terminated their mentoring during the COVID-19 pandemic, mentors were asked the question “Was the mentoring continued during the pandemic?” with the three answer options 1 = “Yes, the mentoring was continued during the pandemic,” 2 = “No, the mentoring was terminated before the pandemic” and 3 = “No, the mentoring was terminated during the time of the pandemic.” Answer options 1 and 3 were transferred to a binary variable, indicating premature match closure. Participants who had chosen answer option 2 were removed from the sample (see above).

### 4.3 Statistical analysis

To test our hypothesized framework, we calculated structural equation models (SEM) with the “lavaan” package (Rosseel, 2012) in the statistical environment R (R Core Team, 2021). All variables were modeled as manifest indicators. As some of the variables were non-normally distributed, we used diagonally least squares estimation procedures (Flora and Curran, 2004) which has been shown to produce more accurate results than ML estimation with ordinal data such as Likert scales and in situations in which there are any non-normally distributed variables (Mîndrilă, 2010). Furthermore, this estimation procedure is especially recommended if the dependent variable is categorial, e.g., binary as our outcome variable premature match closure in our hypothesized model (Steinmetz, 2015). As only a few cases could not be included in the calculation of the SEM due to incomplete data (3 participants, 5%), no biases and loss of power are expected due to missing data (Graham, 2009). To assess the model fit, chi-square statistics, comparative fit indices (CFI), and root-mean-squared errors of approximation (RMSEA) were calculated following the guidelines of Hair et al. (2014). However, we set stricter evaluation criteria as our sample size was small and there were fewer than 12 indicator variables in our model: a good model fit is indicated with a CFI higher than 0.97 and a RMSEA smaller than 0.08. To compare different nested models, we used chi-square difference tests. A non-significant result of the chi-square difference means that the fit of the restricted model is not significantly worse than the fit of the unrestricted model, and the restricted model should be favored (Schermelleh-Engel et al., 2003). To estimate total indirect mediation effects in our final model, confidence intervals were generated via bootstrapping (*N* = 1,000) at 95% confidence intervals.

### 4.4 Results

Table 1 shows means, standard deviations, and reliabilities for all scales of study 1 below the diagonal. Twenty-two of the 98 study participants (22%) indicated that they had closed the relationship with their mentee prematurely during the pandemic.

In the first step, we calculated an extended model of our theoretical model described above. To compare the hypothesized mediation effects with the direct effect of mentors' resources on premature match closure, we included the direct path in addition to the two-mediator serial paths proposed in our initial framework. The model showed a good fit [χ^2^(8) = 1.47, *p* = 0.993, CFI = 1.00; RMSEA = 0.00, 95% CI (0.00, 0.00)]. While the coefficients in the upper mediation path were all significant (mentors' resources ➔ confidence in abilities: β = 0.81, *p* < 0.001, confidence in abilities ➔ helplessness: β = −0.40, *p* = 0.007, helplessness ➔ premature match closure: β = 0.27, *p* = 0.007), there was no significant direct effect of mentors' resources on premature match closure (β = −0.09, *p* = 0.561). The coefficients in the lower path of the model were partly significant (mentors' resources ➔ modifiability and stability beliefs: β = 0.81, *p* < 0.001; modifiability and stability beliefs ➔ adaptive failure response: β = 0.65, *p* < 0.001, adaptive failure response ➔ premature match closure: β = −0.09, *p* = 0.550).

To examine whether the mediators in the upper and the lower path of our model were correlated, we further extended this model in the second step. We modeled the helplessness of mentors and their reactions to failure depending both on their confidence in abilities and modifiability and stability beliefs. Additionally, we allowed the residuals of confidence in abilities and modifiability and stability beliefs to covary, as well as the residuals of helplessness and adaptive failure response. This model also fitted the data very well [χ^2^(4) = 1.01, *p* = 0.908, CFI = 1.00; RMSEA = 0.00, 95% CI (0.00, 0.06)], but the chi-square difference test showed no difference in fit compared to the first, more restricted model [Δχ^2^(4) = 0.45, *p* = 0.978]. This suggests that adding further paths between the mediators does not result in a better model fit. Results of this model revealed four significant path coefficients: the paths directing from mentors' resources to their confidence in abilities (β = 0.75, *p* < 0.001) and their modifiability and stability beliefs (β = 0.80, *p* < 0.001), the path from mentors' beliefs to adaptive failure response (β = 0.74, *p* = 0.045), and the path of helplessness to premature match closure (β = 0.27, *p* = 0.008). These results support that the two mediation paths in our framework are independent.

**Figure 1:**
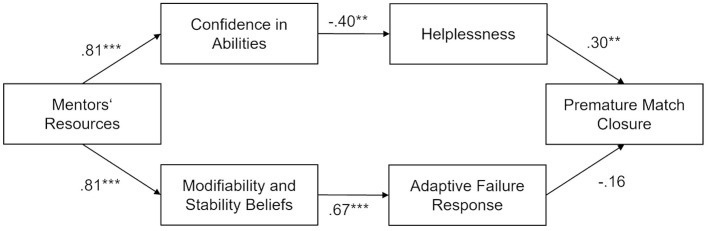
Hypothesized path model from Study 1. The coefficients presented show standardized path estimates. *p < 0.05, **p < 0.01, ***p < 0.001.

In the third step, we tested our hypothesized model with two independent two-serial mediators connecting mentors' resources with premature match closure. The model-fit was good [χ^2^(9) = 1.76, *p* = 0.995, CFI = 1.00; RMSEA = 0.00, 95% CI (0.00, 0.00)]. The non-significant chi-square difference tests comparing this model with the first model (Δχ^2^(1) = 0.30, *p* = 0.586) and the second model [Δχ^2^(5) = 0.75, *p* = 0.980] indicate no worse fit of our hypothesized model and suggest that the direct path from mentors' resources to premature match closure as the paths connecting the mediators should be eliminated. Figure 1 shows the standardized path coefficients, which all turned out to be significant except the path linking adaptive failure response to premature match closure (β = −0.16, *p* = 0.165). Contrary to expectations, the total mediation effects in the model were not significant [upper path: β = −0.10, *p* = 0.060, 95% CI (−0.20, 0.00); lower path: β = −0.09, *p* = 0.179, 95% CI (−0.22, 0.04)]. 13.3 percent of the variance in premature closure was explained by the model.

## 5 Study 2

To cross-validate the results of the first study, we conducted a second study among teachers who were working as personal mentors in the Learning Pathway mentoring program, a program that was established as a part of a German-wide initiative to promote high-achieving and potentially high-achieving pupils funded by the German Federal Ministry of Education and Research (Matthes et al., 2024). In school-based one-on-one mentoring, mentors accompanied individual students who were particularly interested and high-achieving in a specific domain for a period of 3 years. The mentors were teachers who were explicitly trained for their role as mentors and were supported throughout the implementation by university staff with extensive expertise in mentoring.

The mentoring consisted of regular sessions held at school and included the use of accompanying support diagnostics to set goals and plan an individual learning pathway for each student.

### 5.1 Method

We invited 90 mentors who had started mentoring in autumn 2019. After having reminded the mentors to participate, 63 persons completed the survey, representing a 70% response rate. We removed three participants from this sample because they indicated that they had interrupted the mentoring before the pandemic or had not answered whether the mentoring was continued during the pandemic. The final sample consists of 30 female and 30 male mentors spread across 26 schools throughout Germany (*M*_*age*_= 42.80, *SD*_*age*_ = 8.43).

Data was collected in the same period as in Study 1 (October and November 2020). The questionnaire was sent as an editable PDF document via e-mail and collected via e-mail. The structure of the questionnaire and the measures obtained were identical to those of Study 1. Internal consistencies of all scales were acceptable (see Table 1).

### 5.2 Results

In Table 1, means, standard deviations, reliabilities, and intercorrelations for all scales for Study 2 are shown above the diagonal. Mentors in the school-based program differed substantially in their answers compared to mentors in the online mentoring program: mentors in the school-based program had fewer resources during the COVID-19 pandemic [*t*_(156)_ = 4.37, *p* < 0.001, *d* = 0.71], were less confident in their mentoring abilities [*t*_(155)_ = 2.93, *p* = 0.004, *d* = 0.48], and had lower modifiability and stability beliefs [*t*_(156)_ = 3.64, *p* < 0.001, *d* = 0.60]. Twenty-two out of 60 mentors (37%) indicated that their mentoring relationship had been closed prematurely during the pandemic.

We tested the same three models as in Study 1, and the estimation procedure was identical. Four cases had to be excluded from the calculation of SEM due to incomplete data. The first model with a direct path directing from mentors' resources to premature match closure and two indirect paths, each constituting of two mediators in serial, showed a good fit [χ^2^(8) = 4.48, *p* = 0.812, CFI = 1.00; RMSEA = 0.00, 95% CI (0.00, 0.10)]. There were significant positive associations between mentors' resources and their confidence in their mentoring abilities (β = 0.85, *p* < 0.001) as well as modifiability and stability beliefs (β = 0.85, *p* < 0.001). The subsequent paths from confidence in abilities to helplessness (β = −0.71, *p* < 0.001) and mentors' modifiability and stability beliefs to adaptive failure response were significant as well (β = 0.57, *p* < 0.001). Regarding the paths directing to premature match closure, only one significant relation could be found (adaptive failure response ➔ premature match closure: β = −0.29, *p* = 0.019).

The expanded second model with additional interconnections between the mediator variables also fitted the data very well [χ^2^(4) = 0.13, *p* = 0.998, CFI = 1.00; RMSEA = 0.00, 95% CI (0.00, 0.00)], but yielded no better fit than the first model [Δχ^2^(4) = 4.34, *p* = 0.361]. Again, the paths from mentors' resources to their confidence in abilities (β = 0.71, *p* < 0.001) and their implicit beliefs about modifiability and stability (β = 0.68, *p* < 0.001) were significant. Additionally, the path from adaptive failure response to premature match closure (β = −0.26, *p* = 0.046) was significant. There was a significant relation between mentors' confidence in their mentoring abilities and their modifiability and stability beliefs (β = 0.44, *p* < 0.001).

**Figure 2:**
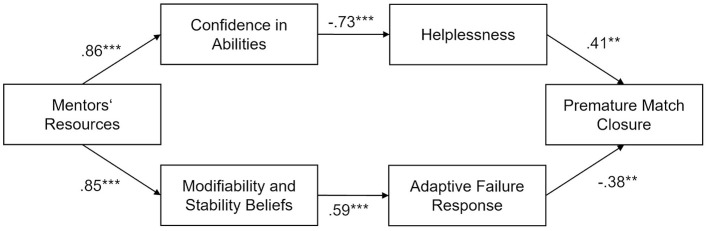
Hypothesized path model from Study 2. The coefficients presented show standardized path estimates. *p < 0.05, **p < 0.01, ***p < 0.001.

Our final model with two-mediator serial paths without interconnections between the mediators showed an excellent model fit as well [χ^2^(9) = 5.07, *p* = 0.828, CFI = 1.00; RMSEA = 0.00, 95% CI (0.00, 0.09)]. Like in Study 1, it fitted the data equally well as the first model [Δχ^2^(1) = 0.59, *p* = 0.442] and the second model [Δχ^2^(5) = 4.94, *p* = 0.423], suggesting that it should be favored due to parsimony. All path coefficients in the upper and the lower mediation paths were significant (see Figure 2). The total mediation effects were significant as well [upper path: β = −0.27, *p* = 0.014, 95% CI (−0.46, −0.05); lower path: β = −0.19, *p* = 0.029, 95% CI (−0.35, −0.02)]. 40.3 percent of the variance in premature match closure was explained by the model.

## 6 Discussion

We conducted two surveys among mentors in two mentoring programs to test a model on the psychological processes that lead to the premature termination of mentoring relationships in dependence on available mentoring resources. We chose to do our research during the first peak of the COVID-19 pandemic in Germany as this created a challenging context in which resources were restricted in many respects.

### 6.1 Relationship between mentoring resources and premature match closure

In both programs, mentoring relationships more often terminated prematurely during the COVID-19 pandemic if mentors reported that they had few resources for their mentoring during this time. This finding supports the assumption that resources play an essential role in implementing mentoring relationships (Stoeger et al., 2021), which has not yet been empirically investigated regarding all the mentoring resources available for mentors. The premature termination of mentoring relationships is a severe problem in mentoring practice, leading to poorer or even negative mentoring outcomes (Grossman and Rhodes, 2002). The study's results highlight the importance of mentors' resources for positive mentoring outcomes in challenging situations.

However, there were differences in available resources and their consequences concerning the development of mentoring relationships between the school-based program, where the mentoring usually takes place in school, and the online mentoring program, where mentors exchange with their mentees exclusively on an online platform. Mentors in the school-based program reported considerably lower mentoring resources, and these were linked more firmly to premature match closure than in the online program, resulting in a higher rate of mentoring relationships ending prematurely. This can be seen as a result of the drastic restrictions at school during the COVID-19 pandemic (school closures, switch to online teaching) and corresponds to other research revealing diverse adverse effects of these restrictions on students (Hammerstein et al., 2021; Deng et al., 2023) and teachers (Robinson et al., 2023). As online mentoring is hardly affected by regulations and social restrictions imposed during the COVID-19 pandemic, mentors experienced less impairment of mentoring resources.

### 6.2 Mediation through confidence in abilities and modifiability and stability beliefs

Although the overall mediation paths in the final models tested in the two studies were only significant among mentors of the school-based program, our analysis supports our hypothesized mediation via two psychological processes at the mentor level. First, in both mentoring programs, mentors' perceived resources during the COVID-19 pandemic were positively related to their confidence in their mentoring abilities. Lower confidence in mentoring abilities was connected to feelings of helplessness, leading to premature match closure during the pandemic. This finding aligns with other studies showing the importance of mentors' self-confidence for mentoring outcomes (Parra et al., 2002; Raposa et al., 2016).

Second, in both programs, mentors' resources were positively related to mentors' beliefs on the modifiability of their deficits and the stability of their abilities regarding their mentoring practice. These beliefs on the nature of their abilities, in turn, were linked to whether they adaptively reacted to failure. Maladaptive failure response was finally linked to the premature termination of mentoring relationships during the COVID-19 pandemic, but only in the school-based program. This shows that beliefs about the nature of abilities which have been associated with motivational, cognitive, and behavioral processes, particularly in learning and achievement situations in previous research (Ziegler and Stoeger, 2010; Burnette et al., 2020, 2013) are also relevant to mentors and their influence on the development of mentoring relationships. Further, we provide additional evidence for the usefulness of the extended framework of implicit personality theories on modifiability and stability to explain individual behavior in challenging situations.

### 6.3 Implications for practice

The study results indicate that it is essential to equip mentors with sufficient resources to achieve positive mentoring outcomes. Preparing mentors through thorough training not only enlarges mentoring competencies but also increases mentors' readiness and perceived confidence in their mentoring abilities (Kupersmidt et al., 2017a). By increasing mentors' internal resources, they might be better prepared for challenging situations in the mentoring, either arising in the interaction with their mentee or in the environment. To master such challenges, it is crucial that mentors further receive ongoing support through the program staff of the mentoring agency. This support might include post-match mentor training and regular mentor monitoring contacts, which help to react immediately if difficulties arise (Garringer et al., 2015). Ongoing monitoring of mentoring relationships, whether through regular contact between program staff members and mentors or by tracking activities, e.g., via an online platform, can indicate deterioration in the mentoring relationship at an early stage and thus contribute to the prevention of premature match closure (DeWit et al., 2016).

If environmental changes accompanied by a limitation of resources put a unique challenge on mentors, specific measures might be helpful. In online focus groups among mentors during the COVID-19 pandemic, Kaufman et al. (2022) asked about mentors' specific needs during that time. The responses indicate that it might be conducive to support mentors in finding suitable ways to connect with their mentees (if face-to-face communication is impaired), establishing support groups for mentors, and providing specific information and resources for mentoring to support mentees during a crisis.

Finally, from the comparison of the two mentoring programs, we conclude that online mentoring is a robust form of mentoring that can alleviate possible adverse effects in challenging times, provided that mentoring is suitably implemented (Stoeger et al., 2021).

### 6.4 Limitations and suggestions for future research

Two limitations should be noted. First, we used cross-sectional retrospective data to examine the sequential mediation model. Although our model is grounded in strong theoretical foundations, the causal relationship between the examined variables cannot fully be acknowledged without an experimental and longitudinal study design. Second, the sample sizes in our studies were relatively small, with 98 and 60 participants. Conducting SEM with small sample sizes results in less statistical power and may cause bias in estimation parameters (Wolf et al., 2013). However, the correlational analysis supports the results of our SEM, which may even be underestimated due to the small sample size. Furthermore, our model held up in two different empirical contexts.

Further research should be initiated to verify the importance of mentors' resources in the mentoring processes indicated in this study by using a longitudinal study design with larger sample sizes in different mentoring settings. It would be interesting to investigate whether the processes uncovered in this study are also triggered in other challenging situations, such as personal crises in mentors' individual lives or a loss of program support. Our study design suggests that the restriction in resources had an influence on mentors' confidence and beliefs, and subsequently led to the premature closure of mentoring relationships within a time period of up to 4 months. As the effects of COVID-19 became apparent very quickly and drastically in everyday life, the effects probably became apparent even earlier. In future longitudinal studies on this topic, the time intervals between measurement points should be selected based on the specific study context.

Furthermore, we propose examining whether the characteristics of the mentoring dyad have an impact on how challenging situations are dealt with. Research suggests that mentoring relationships may be more beneficial and last longer if mentors and mentees share certain characteristics, such as similar interests (DuBois et al., 2011), ethnicity (Raposa et al., 2019a; Blake-Beard et al., 2011), and gender (Blake-Beard et al., 2011). It could be hypothesized that the closeness in the mentoring relationship resulting from similarity may act as a psychological buffer under challenging situations, such as limited communication opportunities, and help the mentoring dyad to continue their mentoring relationship despite external obstacles. Unfortunately, the small sample size of our study did not allow us to analyze these factors.

In addition, it could be investigated how the frequency of meetings between mentors and mentees affects the probability of premature match closure in challenging situations. This might complement previous research, which has shown that mentoring relationships in which mentoring dyads meet or communicate more frequently are likely to last longer (DeWit et al., 2016; Uebler et al., 2023).

## 7 Conclusion

By looking at mentoring from a systemic perspective, we showed how mentors' resources in challenging situations such as the COVID-19 pandemic are related to the premature termination of mentoring relationships as a central indicator of mentoring success. Our analysis of mediating cognitive and behavioral processes at the mentor level confirmed previous results on the importance of mentors' confidence in their mentoring competencies. We demonstrated that mentors' beliefs on the modifiability of their deficits and the stability of their abilities additionally explain mentors' operating in mentoring relationships and their success. Although our studies suggest that online mentoring has the potential to reduce the negative consequences of resource scarcity in the environment, we conclude that good preparation and ongoing support are fundamental for mentors to conduct long-lasting and successful mentoring relationships.

## Data Availability

The raw data supporting the conclusions of this article will be made available by the authors, without undue reservation.
